# Recombinant Protein Production of Earthworm Lumbrokinase for Potential Antithrombotic Application

**DOI:** 10.1155/2013/783971

**Published:** 2013-12-12

**Authors:** Kevin Yueju Wang, Lauren Tull, Edwin Cooper, Nan Wang, Dehu Liu

**Affiliations:** ^1^Department of Natural Sciences, Northeastern State University, Broken Arrow, OK 74014, USA; ^2^Laboratory of Comparative Neuroimmunology, Department of Neurobiology, David Geffen School of Medicine at UCLA University of California Los Angles, Los Angeles, CA 90095-1763, USA; ^3^Biotechnology Research Institute, Chinese Academy of Agricultural Sciences, Beijing 100081, China

## Abstract

Earthworms have been used as a traditional medicine in China, Japan, and other Far East countries for thousands of years. Oral administration of dry earthworm powder is considered as a potent and effective supplement for supporting healthy blood circulation. Lumbrokinases are a group of enzymes that were isolated and purified from different species of earthworms. These enzymes are recognized as fibrinolytic agents that can be used to treat various conditions associated with thrombosis. Many lumbrokinase (LK) genes have been cloned and characterized. Advances in genetic technology have provided the ability to produce recombinant LK and have made it feasible to purify a single lumbrokinase enzyme for potential antithrombotic application. In this review, we focus on expression systems that can be used for lumbrokinase production. In particular, the advantages of using a transgenic plant system to produce edible lumbrokinase are described.

## 1. Introduction

**Figure 1:**
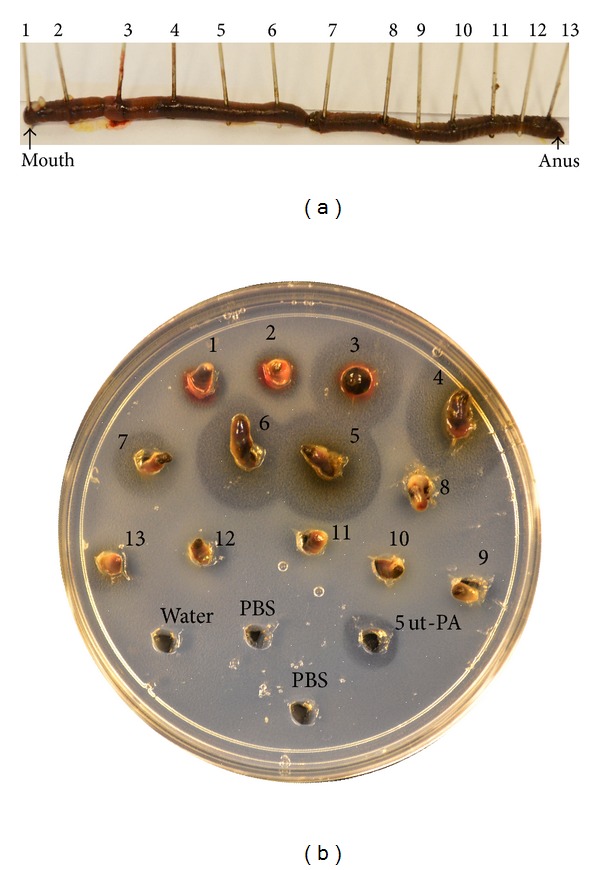
Earthworms exhibit strong fibrinolysis. (a) An earthworm, *Eisenia fetida*, was collected locally (Tulsa, OK, USA) washed with deionized water and sectioned in 13 pieces. (b) The sections were applied directly to a petri dish plate containing artificial fibrin and incubated at 37°C overnight. Human tissue plasminogen (t-PA) (5 units) was used as a positive control. Deionized water and phosphate buffered saline (PBS) served as negative controls. Bigger lysis halos (3–7) indicated higher fibrinolytic activity regions. This method was described by Mihara et al. [10].

Earthworms, which are also called Dilong (Earth Dragon) in Chinese, have been used as a traditional medicine and food resource in China, Japan, and other Far East countries for thousands of years [1–3]. Earthworms contain many compounds with potential medicinal properties and have been administrated to treat inflammatory, hematological, oxidative, and nerve disease [4–6]. Earthworms also have antimicrobial, antiviral, and anticancer properties [7]. Among many properties, earthworms also exhibit fibrinolytic activity [8–11]. The pharyngeal region, crop, gizzard, clitellum, and intestine secret an enzyme that plays a role in dissolving fibrin [9, 10] (Figure 1). Ground-up earthworm powder has been used as oral administration to support circulatory health and treat blood diseases [9].

**Figure 2:**
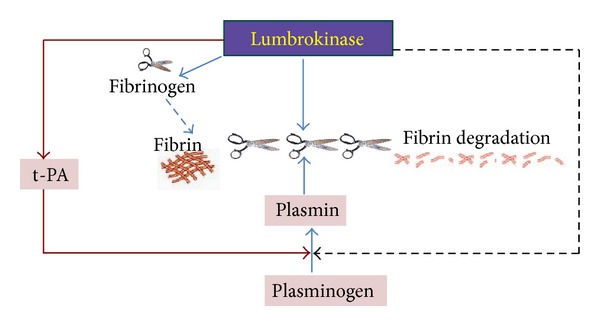
Lumbrokinase mechanism of action. LK dissolves the fibrin itself or increases native t-PA activity to dissolve fibrin clots [15, PLoS ONE].

In 1991, Dr. Mihara and other scientists in Japan successfully extracted and characterized a group of fibrinolytic enzymes from the earthworm species, *Lumbricus rubellus* [10]. These enzymes are capable of degrading both plasminogen-rich and plasminogen-free fibrin. The enzymes were collectively named lumbrokinase (LK) after the genus name for earthworm, *Lumbricus*. Thrombolytic agents typically used to dissolve clots are urokinase (u-PA), streptokinase, and tissue plasminogen activator (t-PA). These drugs, however, are not specific for fibrin and have adverse and dangerous side effects including severe bleeding and heavy blood loss which may result in death [11, 12]. In contrast, LK is very specific to fibrin as a substrate and it does not cause excessive bleeding [13, 14]. It can dissolve the fibrin itself or convert plasminogen to plasmin by inducing endogenous t-PA activity to dissolve fibrin clots [10, 14, 15] (Figure 2: LK mechanism of action).

LK has shown therapeutic promise for use in dissolving clots, lowering whole blood viscosity, and reducing platelet aggregation. It has not shown any adverse effects on the functions of the nervous system, respiratory system, cardiovascular vessels, or the liver and kidney [2, 7]. Currently, LKs are widely used clinically as a thrombolytic agent in China to treat cerebral infarction, coronary heart disease, pulmonary heart disease, deep vein thrombosis. angina pectoris, diabetes, and cerebral infarction. In Japan, Korea and also in North American countries such as Canada and the United States, LK has been used as oral supplement to support and maintain healthy cardiovascular function.

## 2. Extraction and Isolation of LKs

LKs have been mainly isolated from *L. rubellus* and *Eisenia fetida*. Some reports refer to LKs as earthworm fibrinolytic enzymes (EFE) or earthworm powder enzymes (EPE). Some proteases have been named after the Latin binomial for the earthworm species from which it was derived [9]. For example, a protease obtained from *E. fetida* is called *E. fetida protease (Efp)* [9]. Mihara et al. [10] extracted six fibrinolytic enzymes (F-1-0, F-1-1, F-I-2, F-II, F-III-1, and F-III-2) from *L. rubellus*. Seven fibrinolytic enzymes were purified from *E. fetida* by Zhou et al. in 1988 [16]. Isozymes of LK have also been isolated from *L. bimastus* [17] and *E. Andrei* [18]. LKs can maintain activity under both acidic and basic conditions. They have a wide pH range (1-11). Their protein molecular mass is between 20 to 35 kDa and the isoelectric points (pI) range from 3 to 5. Some LKs are also resistant to high temperatures (up to 60°C) [9]. Since each fibrinolytic enzyme was independently isolated and named by different research groups, the same enzyme may have multiple names. Therefore, the total number of LKs is not clear [9]. LK nomenclature needs to be standardized based on proteinase function, property, and the source.

Conventional methods of LK extraction and purification from earthworm are complicated and time consuming. The process consists of multiple steps that include ammonium sulfate precipitation and filtration, ion exchange chromatography, hydrophobic interaction chromatography, and affinity chromatography [10, 19]. Since the molecular range of LK is relatively narrow (20–35 kDa), it is very hard to isolate and purify a single LK protein with conventional methods. Thus, LK products usually contain multiple enzyme components. The use of different extraction and purification procedures will also result in a final product that varies in LK composition. Thus, the level of fibrinolytic activity may also vary. The final product may also contain other earthworm contaminants that can induce adverse side effects, such as an upset stomach or vomiting [20]. Therefore, researchers have used recombinant DNA technology for the expression and characterization of a single LK protein to assess its potential for clinical application.

## 3. LK Gene Cloning and Analysis

**Figure 3:**
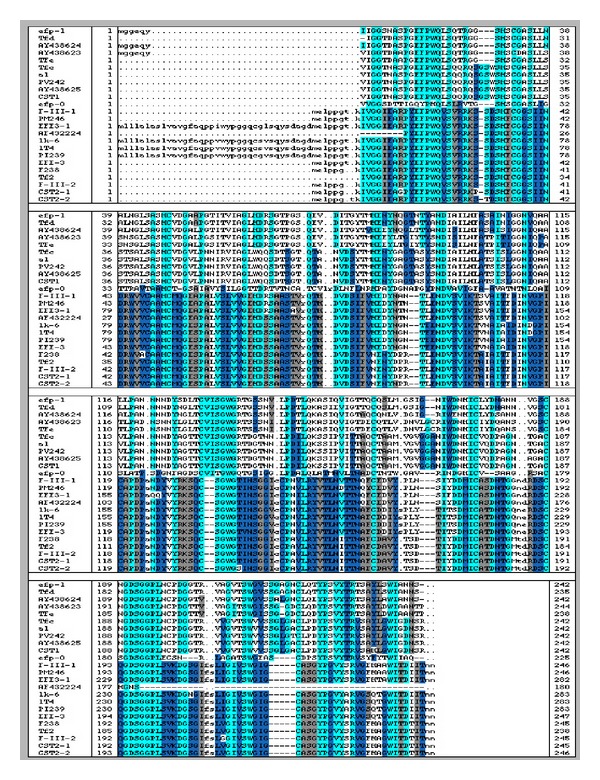
Alignment of 24 LK amino acid sequences. All sequences were obtained from GenBank. Identical residues are highlighted with the same color.

To date, 24 lumbrokinase gene sequences are publicly available at NCBI GenBank (Table 1). Amino acid sequence alignment indicates that some LK genes are highly related (Figure 3). For example, PI239 shares 99% amino acid similarity with 1T4, EFE-3, and lk-6 (F6) [9]. CST1 exhibits the highest level of sequence identity (99%) with the enzyme, PV242, and AF109648 as well as TFc, a1, and AY438625 [15]. The high level of similarity in amino acid sequence among LKs indicates that some LKs have a comparatively recent common ancestor [9, 15]. The phylogenetic analysis also indicates that most LK genes are closely related to each other [9, 15]. The differences in LK protein sequences between species may be the result of the diverse habitats occupied and food resources utilized by the different species of earthworm [9, 21]. Current evidence suggests that mostly *Eisenia* possesses lumbrokinase (Table 1). Their habitats are different from *Lumbricus* terrestris. The amino acid sequence of efp-0 has the lowest identity (25–41%) with all other reported LKs, indicating that it may have evolved independently.

**Figure 4:**
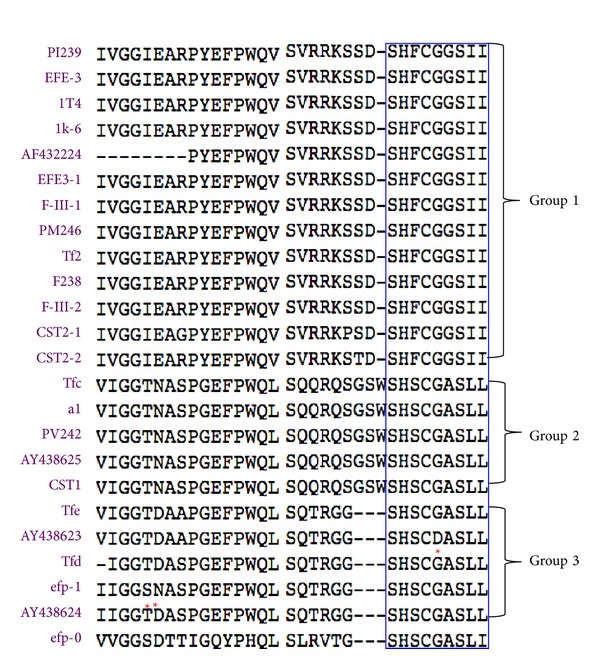
N-terminal sequence alignment of LKs. Except efp-0, the other 23 LKs were placed in three groups based on their N-terminal amino acid sequence. The conserved residues, S-H-S-C-G-A-S-L-L, are boxed. *indicates an exception.

An analysis of the N-terminal amino acid sequence of LK proteins reveals a high degree of identity with only one or two differences in amino acid (Figure 4). The conserved region at the N-terminal end of LKs suggests that it plays an important role in the activity of this enzyme in targeting and degrading fibrin clots [15, 22]. Interestingly, even efp-0, which has less identity to the other LKs, shares the same amino acid sequence, S-H-S-C-G-A-S-L-I, in the N-terminal region of the protein, which further suggests that this region may be very critical for the fibrinolytic properties of LKs. Further molecular studies, utilizing deletion or mutation of specific bases in this part of the gene, are needed to clarify the biological function of this region. LKs share common features to other fibrinolytic proteases, such as t-PA, u-PA, or vampire bat plasminogen activator *α*1 (DSPA*α*1) [9, 15, 22]. They also show similarity to mammalian serine proteases. The catalytic amino acid residues of LKs are very conserved. For example, the catalytic triad, three catalytic subsites, and the primary substrate specificity determinants of t-PA and u-PA are conserved in LK PI239 [22]. Our own analysis found that CST1 contains a catalytic triad, pocket, and substrate recognition sites similar to tPA, uPA, and DSPA*α*1. The amino acids, Ser^209^ and Trp^210^, of CST1 match the S1 and S2 subsites of tPA, uPA, and DSPA*α*1 [15]. These sites play an important role in the catalysis and cleavage of peptide bonds and degradation of fibrin clots [15, 22]. Thus, the conserved features of LKs explain the mechanism of fibrinolytic activity.

## 4. Engineering LKs for Potential Medical Application

Since it is easier and more straightforward to obtain repeatable biological results when evaluating single compounds rather than complex mixtures, most of the drugs approved by FDA are single chemical entities. Similar to most traditional medicines, LKs present challenges in the design of protocols to meet FDA regulations. As mentioned, conventional extraction and purification methods are tedious and have limitations in their ability to isolate and purify a single LK protein from earthworms [10, 16, 19]. Current LK products do not produce consistent thrombolytic results. Therefore, recombinant technology that would allow for the expression of a single LK gene would greatly assist the ability to obtain data for evaluating pharmaceutical safety and efficacy standards.

Sequences for 24 LK genes have been deposited in GenBank (Table 1). Only a few of these genes have been successfully expressed and characterized in *E. coli* [15, 17, 23, 25, 26, 29], goat mammary glands [20], yeast *Pichia pastoris* [21, 22, 24, 28, 30], or plants [27]. Over the past decade, researchers have tried to produce LKs via recombinant technology; however, the majority of studies have reported that, for undetermined reasons, recombinant LKs are either not expressed or do not exhibit fibrinolytic activity. In 2001, Sugimoto and Nakajima [24] cloned two genes encoding the LKs, F-III-2, and F-III-1. Only F-III-2 was expressed in *P. pastoris* and characterized. The secreted recombinant F-III-2 protease was able to dissolve artificial fibrin. The F-III-2 cDNA, with or without a native signal peptide sequence, was further studied using an *E. coli* expression system. Results indicated that *E. coli* could not recognize the native signal peptide of F-III-2 [25]. Therefore, no significant fibrinolytic activity was observed, even though the gene was expressed [25]. Ko et al. [31] cloned and expressed a fibrinolytic enzyme gene from *E. andrei* tobacco chloroplasts. The biological function of recombinant enzyme, however, was not reported.

The development of an animal cell system for lumbrokinase expression was led by Hu and colleagues [20]. Both the wild-type and codon-optimized EFE3-1 gene were expressed in lactating goat mammary glands and characterized. The fibrinolytic activity (550,000 ± 21,600 tPA u/L) of the codon-optimized gene was twice that of the wild-type gene (215,000 ± 13,200 tPA u/L). These results indicate that codon usage bias in different species is very important for LK expression. When the same vectors were transiently transfected in other mammalian cells, such as baby hamster kidney (BHK)-21, Chinese hamster ovary (CH), Vero cells, Madin-Derby canine kidney (MGCK), and COS-7, no lumbrokinase activity was detected. The reason for the lack of fibrinolytic activity is unclear and warrants further investigation.

Due to its ease of handling and rapid growth, the *E. coli* expression system has the potential to produce high yields of LK protein at a low cost. The recombinant LKs produced in *E. coli*, however, are packaged as an inclusion body [15, 17, 23, 25, 26, 29]. As a result, a renaturation process is necessary to recover and reconstitute enzyme activity. Since prokaryotic cells are not capable of performing posttranslational modifications [32], *E. coli* may not be able to express eukaryotic LK proteins with proper folding, processing, and glycosylation. Yeast expression systems utilizing *P. pastoris* may be a better option for producing LK proteins since yeast can accomplish eukaryotic posttranslational modification of recombinant proteins [33]. Even though several LKs have been successfully expressed and characterized with yeast system [21, 22, 24, 28, 30], the glycosylated pattern of the recombinant LK protein has not been investigated. Studies have shown that LKs contain glycan chains [34, 35]. Wu and colleagues [34] isolated eight LKs from *E. fetida*. Glycan measurement showed that all eight proteases were glycoprotein with different carbohydrate contents. The glycosylation of LKs might play important role in LKs' fibrinolytic activity, stability, and proteolysis resistance. Optimized high density fermentation of engineered *P. pastoris* produced 0.1784 g/L of lumbrokinase PI239 in the supernatant [36]. However, the use of methanol to activate lumbrokinase gene expression in the yeast represents a safety concern. Therefore, the production of functional LKs in transgenic plants could represent an attractive alternative.

## 5. Plant-Derived LK Proteins

Genetically modified plants have been developed commercially for the past twenty years [37]. Plants have emerged as a convenient and economic alternative to expression systems that utilize bacteria, yeast, or cultured mammalian cells for the production of pharmaceuticals [37–40]. Plants have the machinery necessary for posttranslational modifications that are necessary to achieve protein stability and bioactivity. The protein synthesis pathway in plants is also very similar to animal cells. The cost of producing pharmaceutical protein in a transgenic plant system is estimated to be much cheaper than using mammalian cell cultures and microbial fermentation systems. For example, computed plant-derived single-dose Hepatitis B vaccine (HBV) will save 62% to 90% depending on the locations of facilities (the United State, Korea, or India) comparing to the yeast-derived vaccine [41]. More importantly, plants are not the host of human pathogens. Therefore, recombinant protein from plants is less likely to transmit disease causing agents to human beings [42].

**Figure 5:**
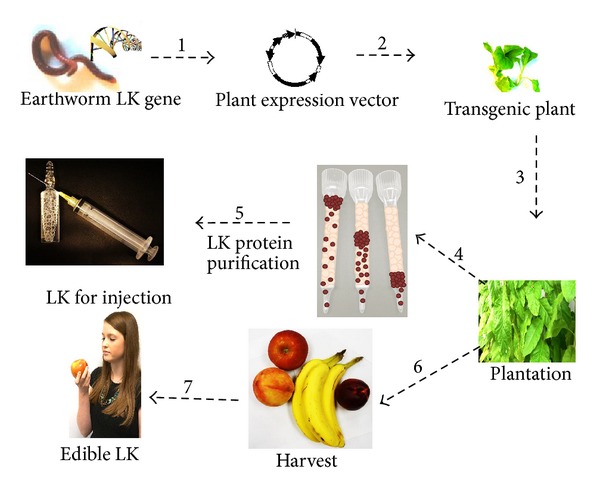
Using transgenic plants for pharmaceutical protein production. A candidate LK gene is introduced into a plant expression vector (1) and integrated into a transgenic plant (2). Transgenic plants are grown (3) and then harvested for protein purification (4) which can then be administered by injection (5) or used as a vegetable or fruit (6) and eaten as a food with therapeutic properties (7).

LKs are very good candidates for recombinant production in a plant expression system. Unlike most proteins, LKs can resist low pH acidic conditions [9, 10]. They can also be absorbed in the gastrointestinal tract intact and retain activity [43–45]. Even though the intact LKs were detected after the intestinal absorption, the mechanism of how LKs could transport into blood is still uncertain. Authors [43, 45] suggested that LKs might cross the cell membrane by exocytosis and enter the blood stream to dissolve clots. N-terminal sequences of the majority LKs are rich in hydrophobic amino acid residues, which may play an important role in the resistance to degradation in the gastrointestinal tract [43]. In a plant expression system, LKs could be expressed in a vegetable or fruit and consumed as pharmaceutical agent directly as food, which would eliminate the need for extraction and purification. LKs could also be expressed in specific tissues like seeds that would allow for downstream processing and purification for oral administration or injection (Figure 5).

To date, only two studies have reported the expression of LKs in plant systems. In 2009, Ko et al. [31] introduced a lumbrokinase gene into tobacco chloroplasts. Their results indicated a stable integration of the LK gene into the tobacco plastid genome and the expression level of the recombinant protein was confirmed by Western blot analysis. Even though fibrinolytic activity was not assessed in this study, it demonstrated that overexpression of LKs in a plant system was feasible. In 2013, Guan et al. [27] produced biologically active EFE3-1 in sunflower seed using the seed-specific promoter, *napA*. A significant antithrombus effect was observed in mice that were fed kernels of transgenic seed. This study demonstrates that plant systems represent an attractive and promising option for the production of therapeutic LKs.

## 6. Conclusions

LKs, in the form of dry earthworm powder, have been widely used clinically in China as an antithrombotic agent. LK capsules have also been used as a health supplement in various countries, including Japan, Korea, Canada, and United States, for supporting circulatory health. The multiple components found in LK products derived from earthworm powders, however, represent a significant barrier to the approval and use of LKs as a pharmaceutical product. Twenty-four LK genes have been cloned and sequenced and it is likely that additional LK genes will be identified and cloned in the future. Since it is very difficult to isolate and purify a single LK protein from earthworms, the production of LKs using recombinant technologies is essential. Optimization of LK gene codons may be a good strategy to increase LK protein. Additional studies are needed to clarify why some LK genes are capable of being expressed in cell culture systems but without fibrinolytic activity. Research is also required to investigate the structure and function of different LKs. Since plants provide a convenient and inexpensive transformation platform for the production of recombinant proteins, we anticipate that more LKs will be expressed and tested in transgenic plants. In addition to the limited yield and slow process of the stably transformed plant system, transient expression in plants using replicating viral vectors can provide high-yield production capacity for pharmaceutical proteins within several days [46–48]. We transient express several fibrinolytic enzymes in plants by a single-vector DNA replicon system (kindly provided by Dr. Hugh Mason, Arizona State University) and the results are promising (data in preparation). In conclusion, plant expression systems represent a promising alternative for the production of LKs for both oral ingestion and injection.

## Figures and Tables

**Table 1:** Lumbrokinase gene clones, expression, and characterization.

Earthworm	Lumbrokinase	GenBank no.	Expression host(s)	Characterization references
*L. bimastus *	PI239	AF433650	*P. pastoris* *E. coli *	Ge et al., 2005 [22] Xu et al., 2010 [23]
	PM246	AY187629	*P. pastoris *	Hu et al., 2005 [21]
	PV242	AF109648	*E. coli *	Xu et al., 2002 [17]

*L. rubellus *	F-III-1	AB045720		
	F-III-2	AB045719	*P. pastoris* *E. coli *	Sugimoto and Nakajima 2001 [24] Li et al., 2008 [25]
	1T4	U25643		
	lk-6 (F6)	AF304199	*E. coli *	Cho et al., 2004 [26]
	EFE3-1	U25648 (AY327442)*	Goat mammary glands Sunflower plant	Hu et al., 2004 [20] Guan et al., 2013 [27]

*E. fetida *	CST1	AY840996	*E. coli *	Li et al., 2012 [15]
	CST2-2	AY684712		
	CST2-1	AY684711		
	TFe	EU167737		
	Tfd	EU167736		
	Tfc	EU167735		
	Tf2	EU167734		
	F238	DQ202401	*P. pastoris *	Zhao et al., 2006 [28]
	efp-1	DQ418454		
	efp-0	DQ836917		
	a1	AF393512		
	EFE-3	AY438622	*E. coli* *P. pastoris *	Dong et al., 2004 [29] Yuan et al., 2006 [30]
		AF432224		
		AY438624		
		AY438623		
		AY438625		

*(AY327442) was codon optimized from U25648.
